# Supplementary figures and images for: Ability of clinical data to predict readmission in Child and Adolescent Mental Health Services
Source: PeerJ Comput Sci. 2024 Oct 18;10:e2367. doi: 10.7717/peerj-cs.2367 (PMC11622991; doi:10.7717/peerj-cs.2367)

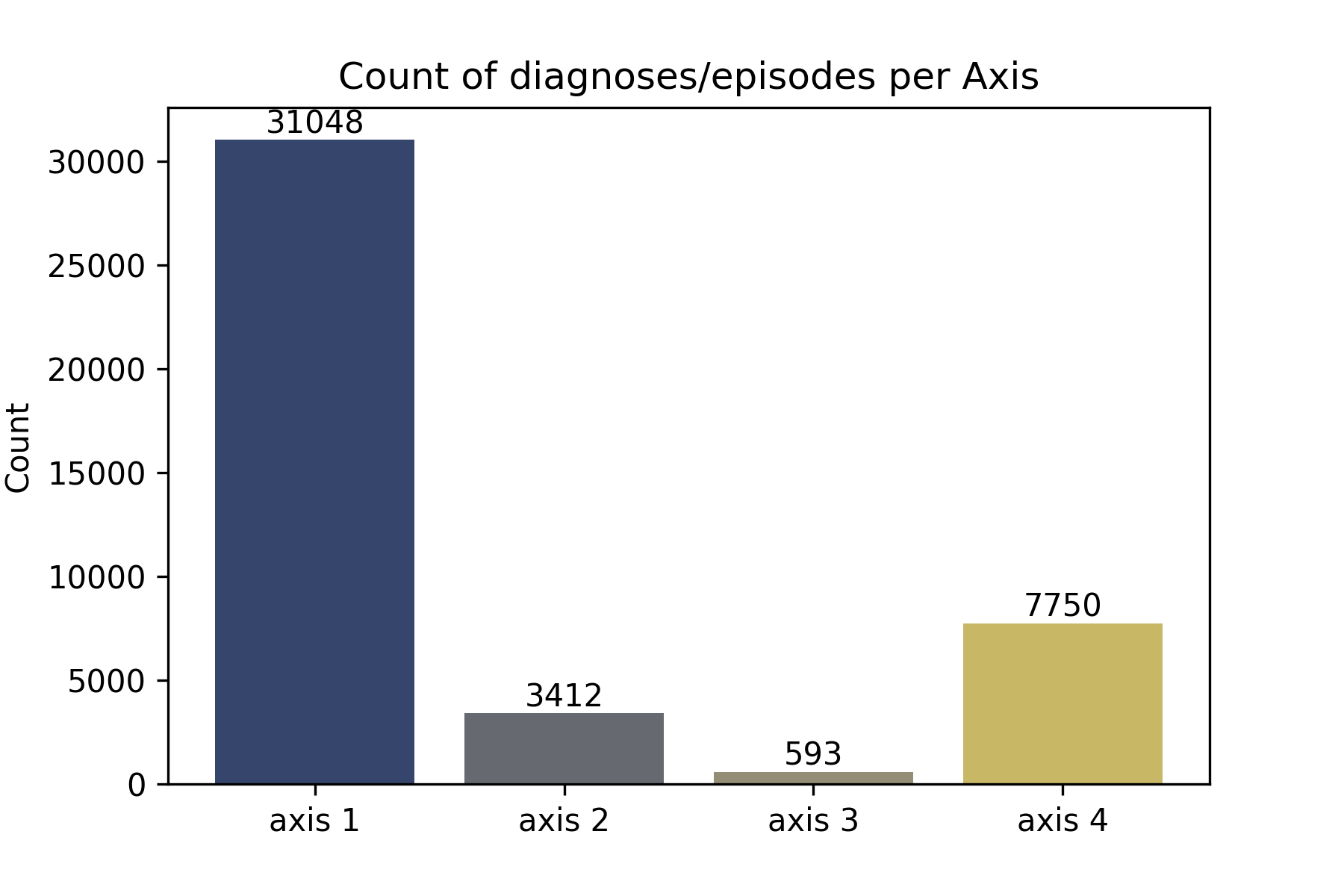

Supplement: Supplemental Information 1 [file peerj-cs-10-2367-s001.png]

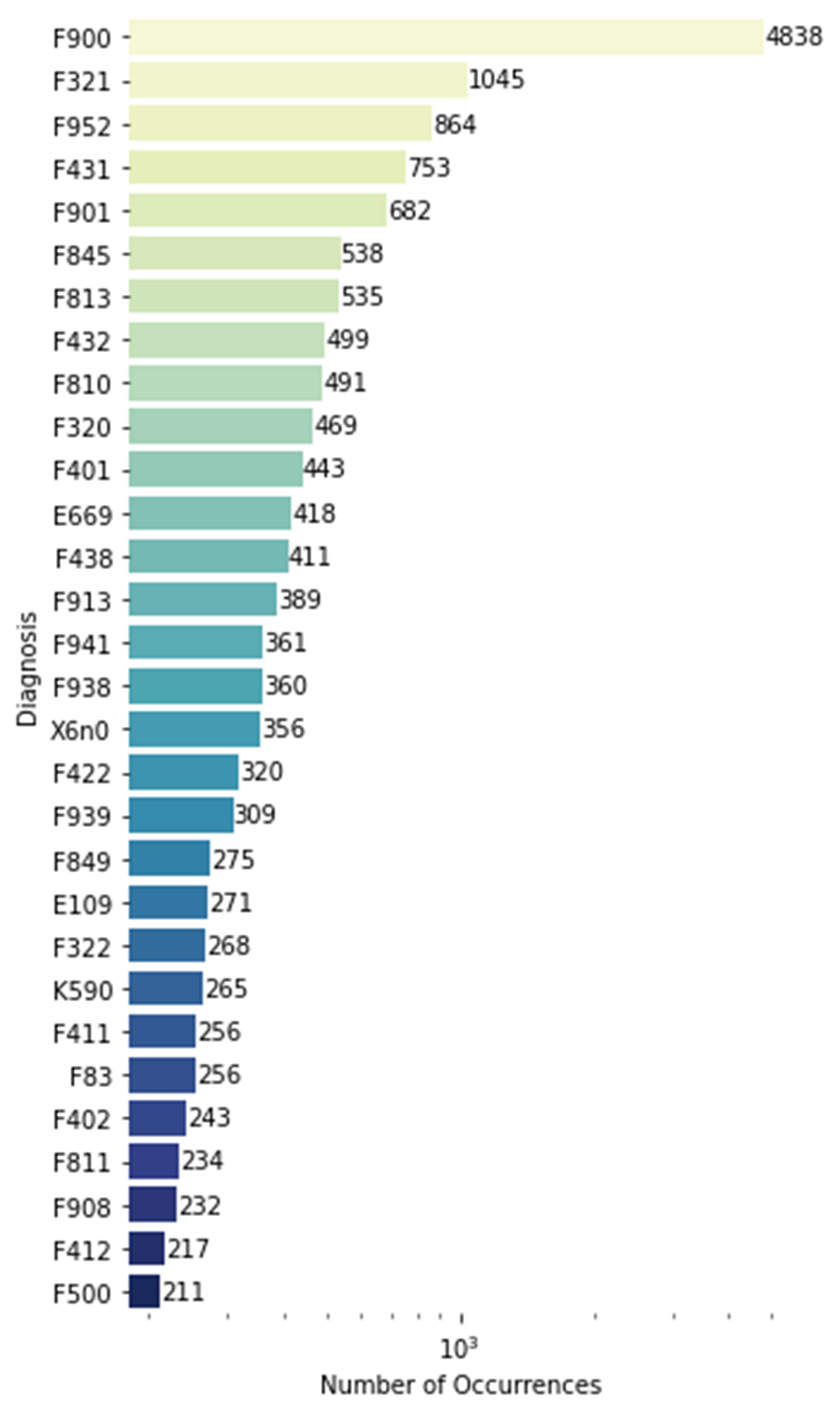

Supplement: Supplemental Information 2 [file peerj-cs-10-2367-s002.png]

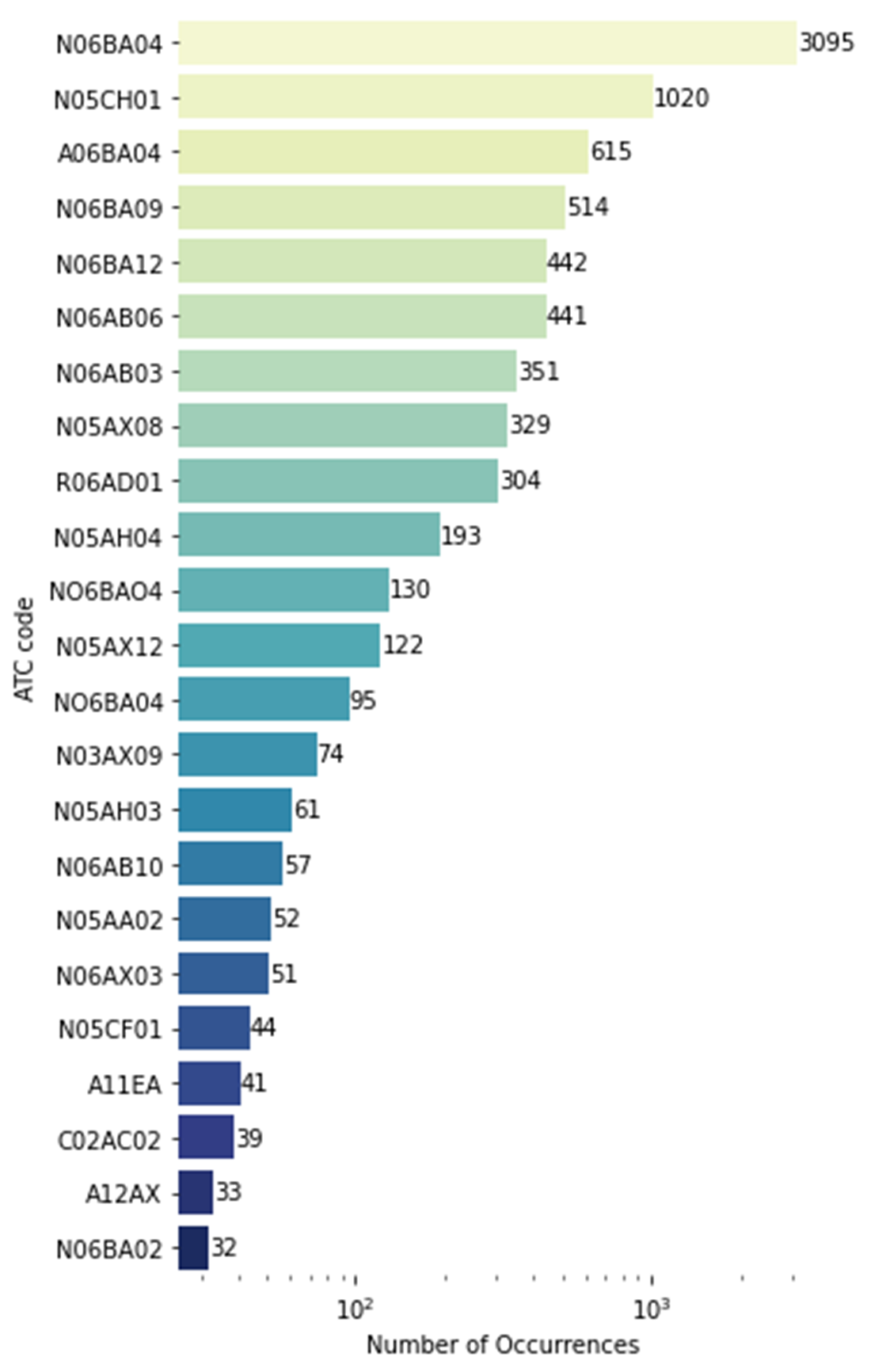

Supplement: Supplemental Information 3 [file peerj-cs-10-2367-s003.png]

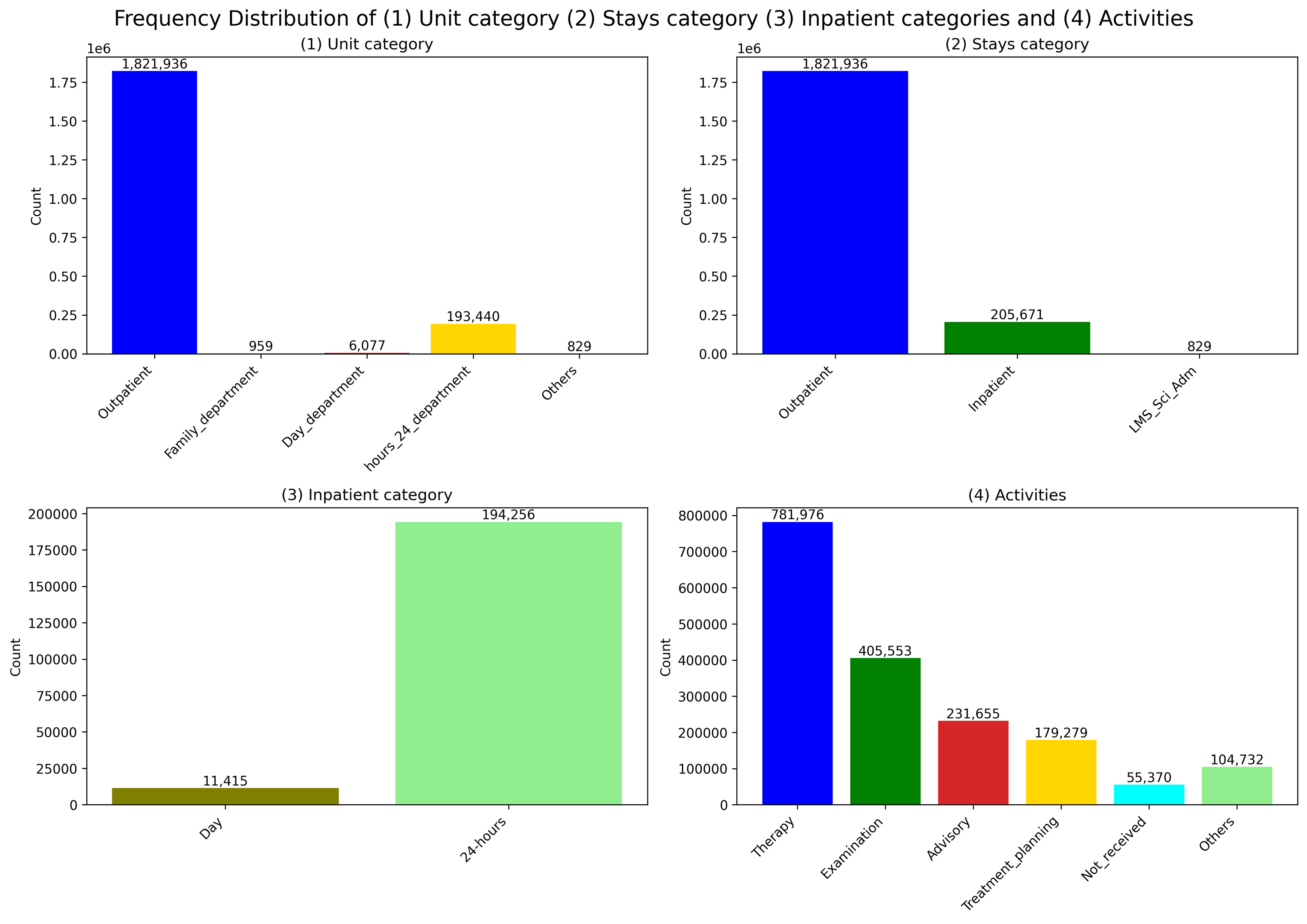

Supplement: Supplemental Information 4 [file peerj-cs-10-2367-s004.png]

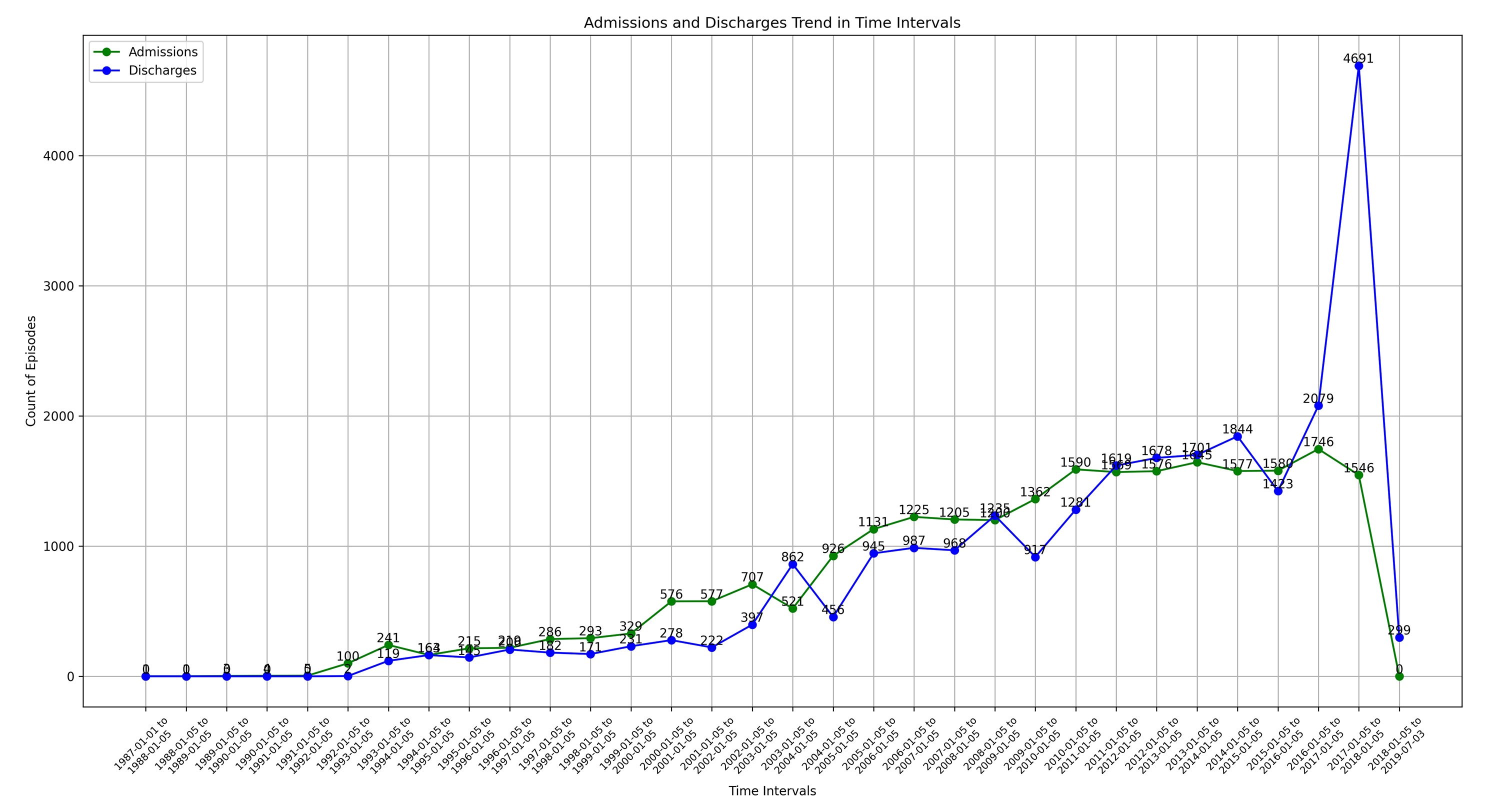

Supplement: Supplemental Information 5 [file peerj-cs-10-2367-s005.png]

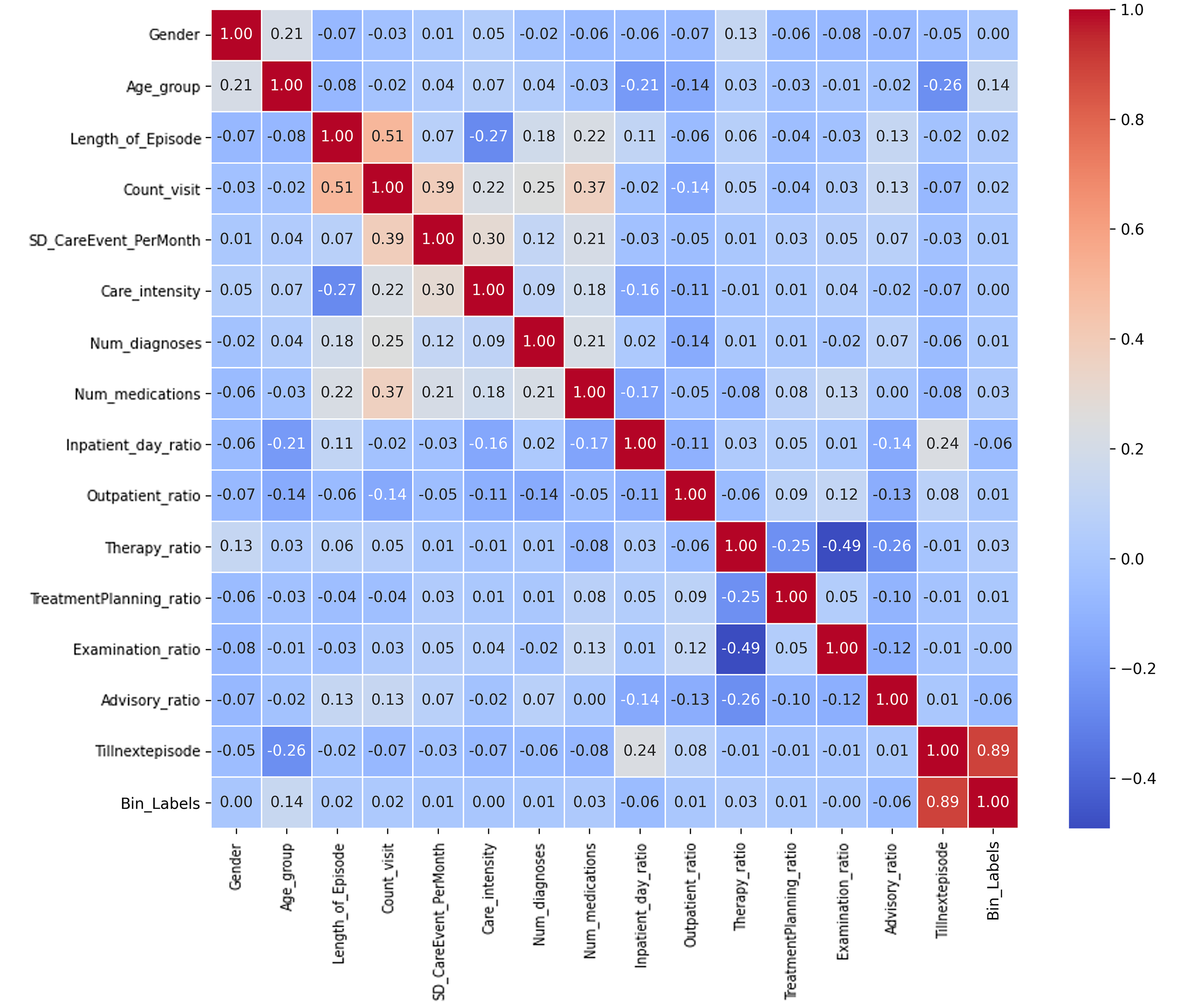

Supplement: Supplemental Information 6 [file peerj-cs-10-2367-s006.png]

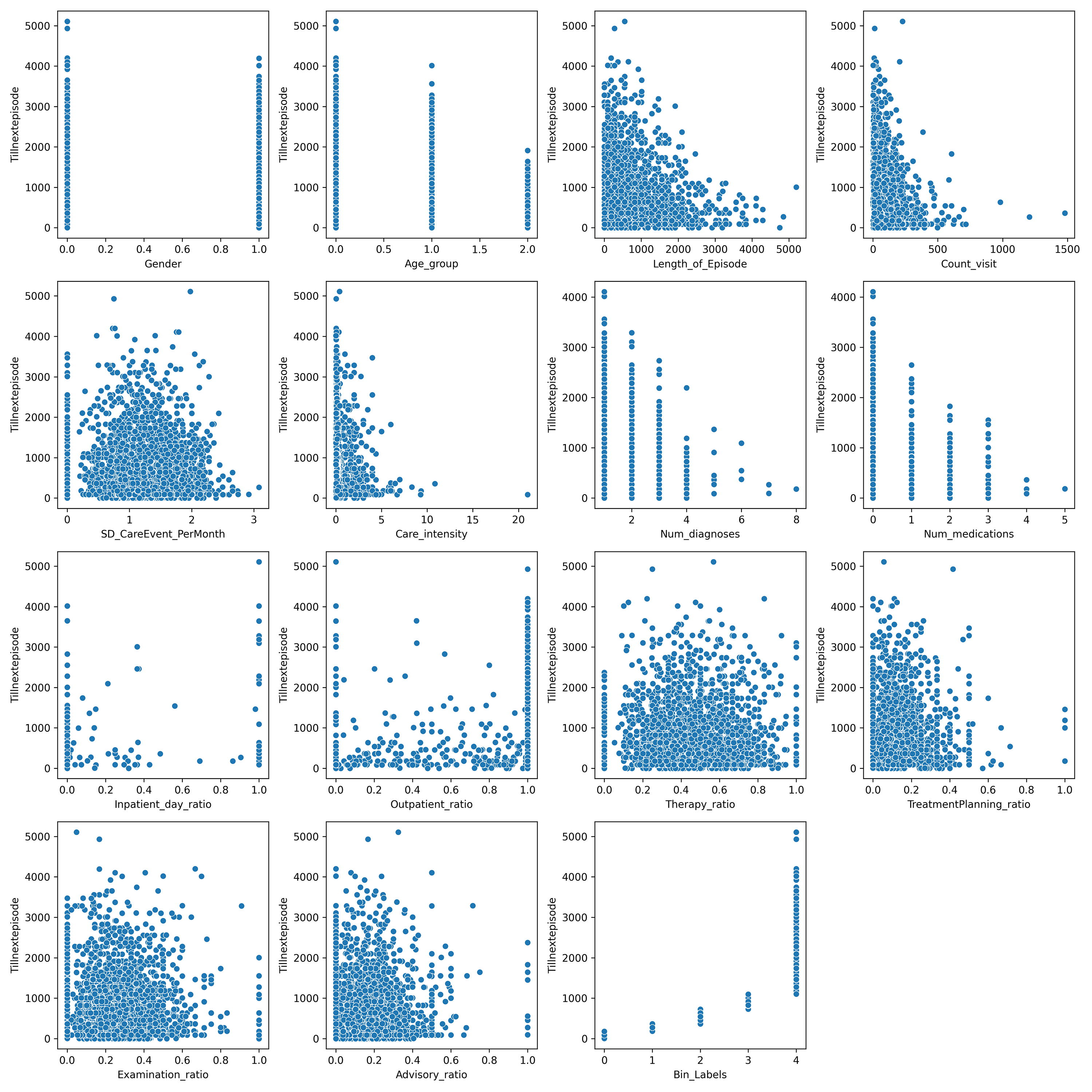

Supplement: Supplemental Information 7 [file peerj-cs-10-2367-s007.png]

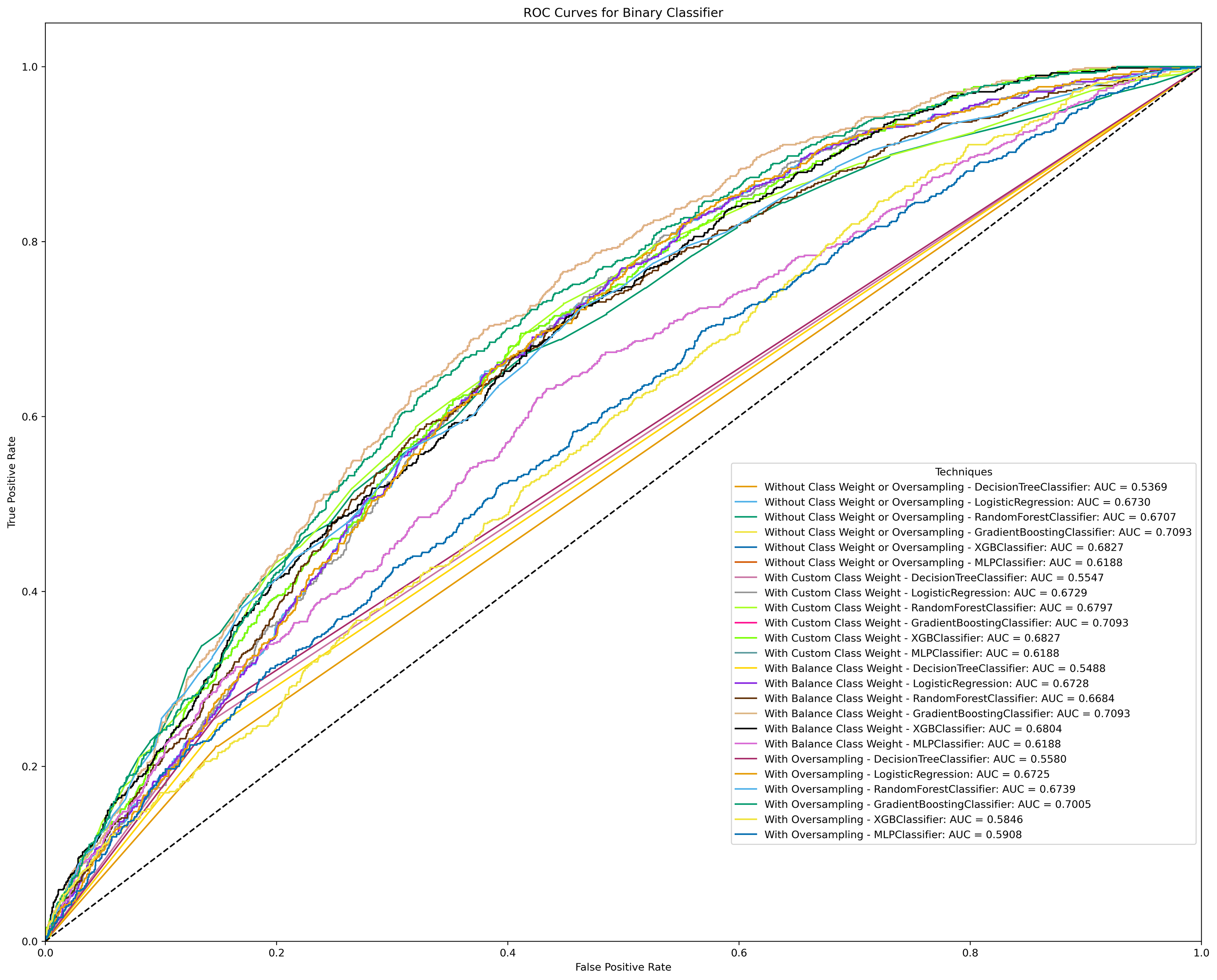

Supplement: Supplemental Information 8 [file peerj-cs-10-2367-s008.png]

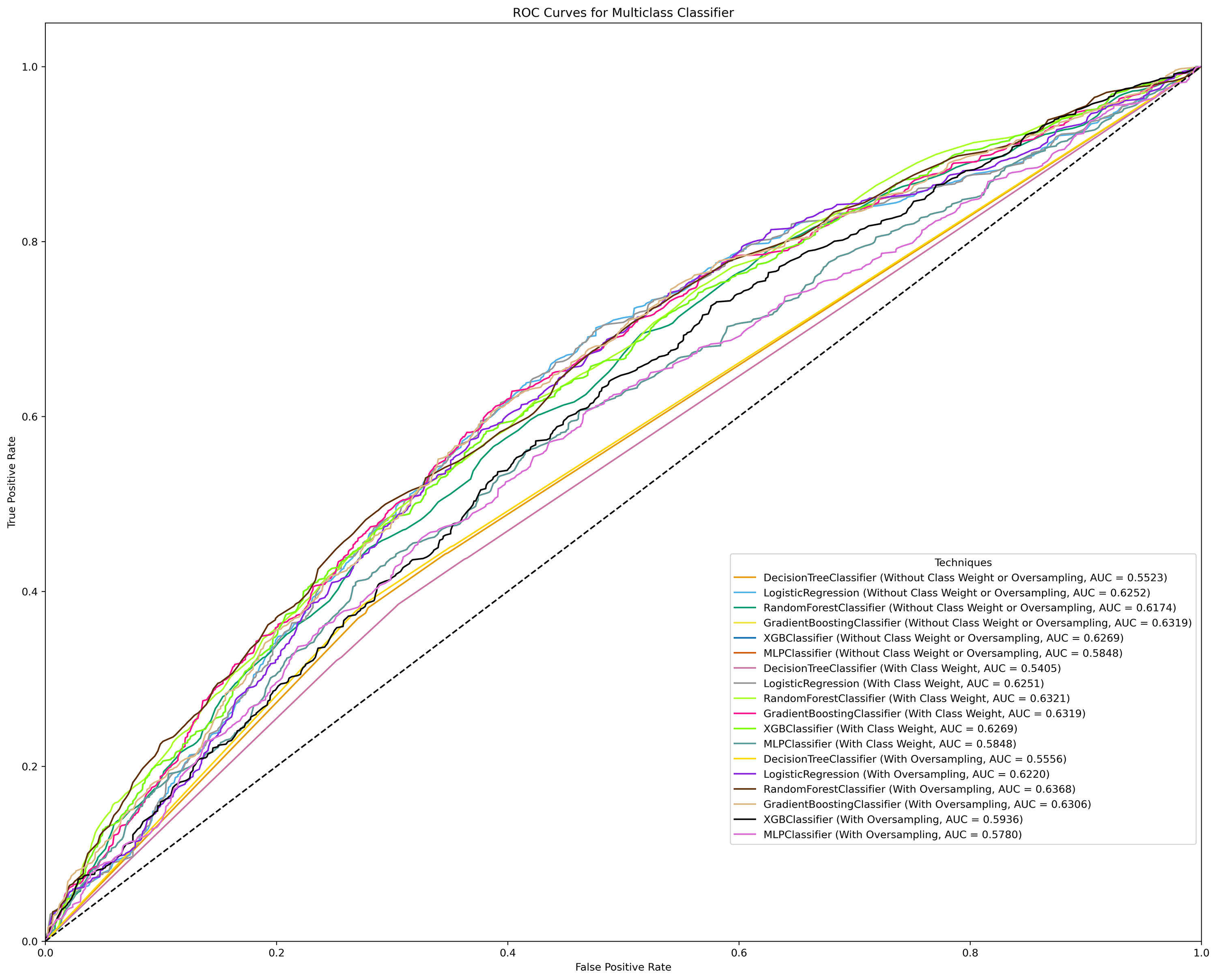

Supplement: Supplemental Information 9 [file peerj-cs-10-2367-s009.png]

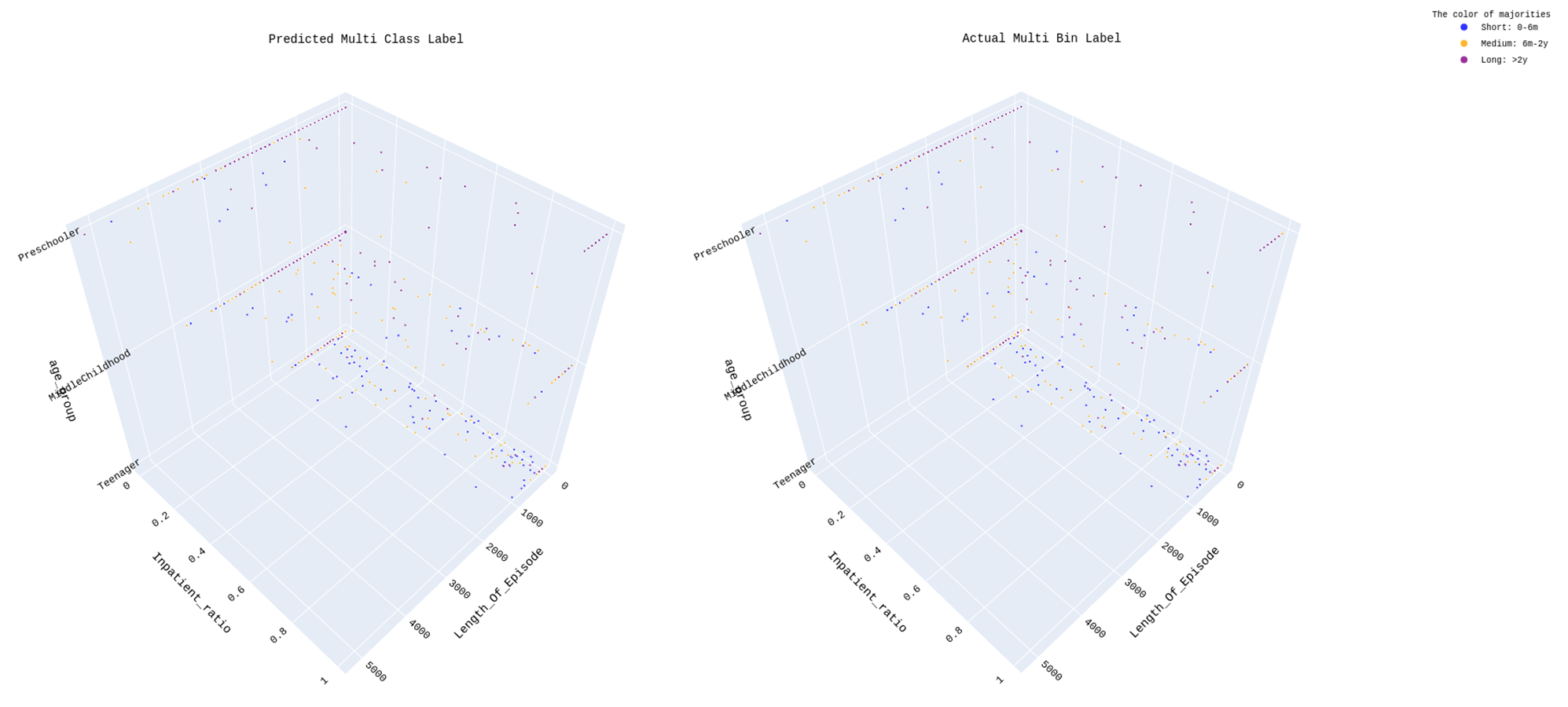

Supplement: Supplemental Information 10 [file peerj-cs-10-2367-s010.png]

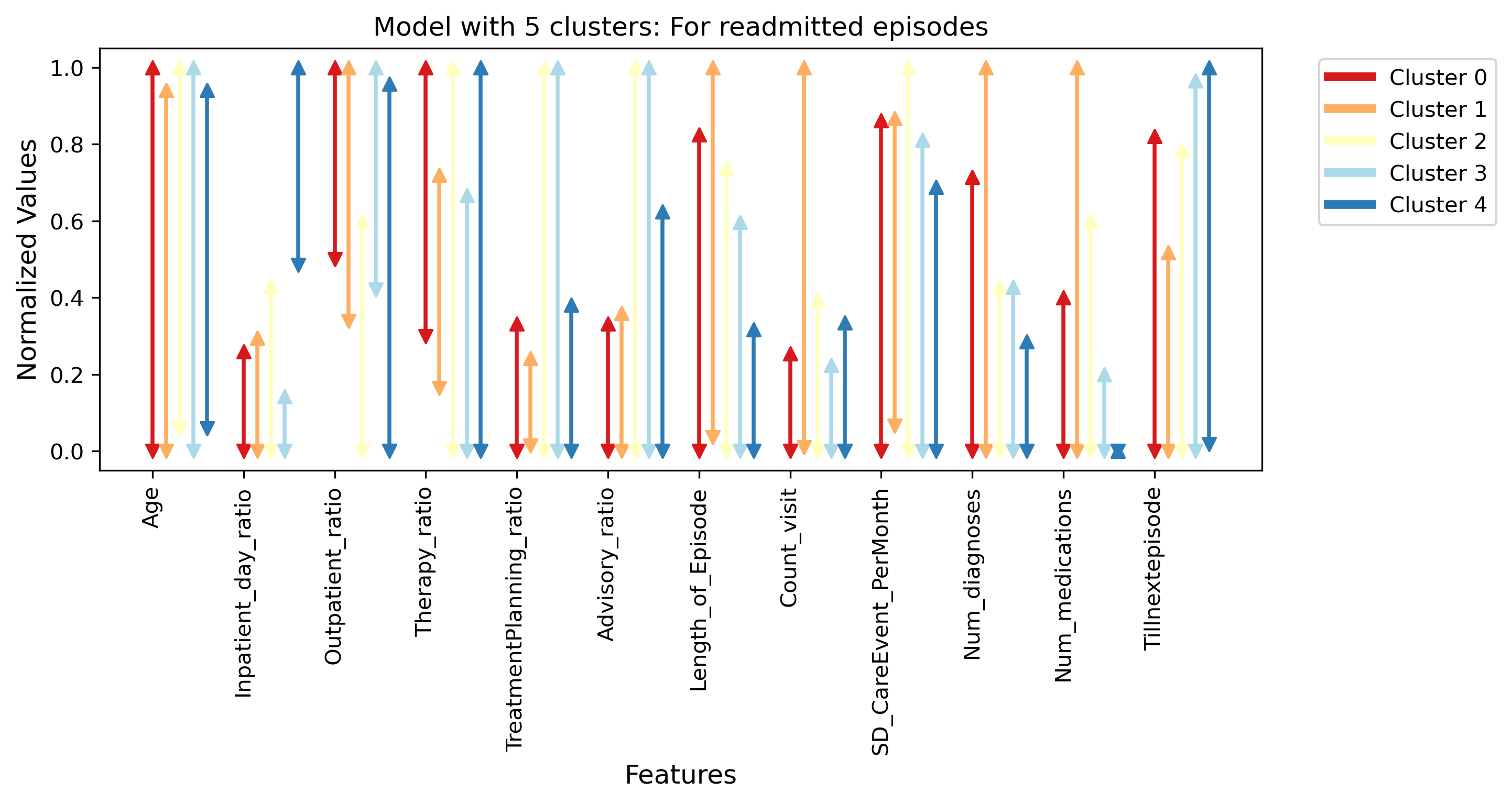

Supplement: Supplemental Information 11 [file peerj-cs-10-2367-s011.png]

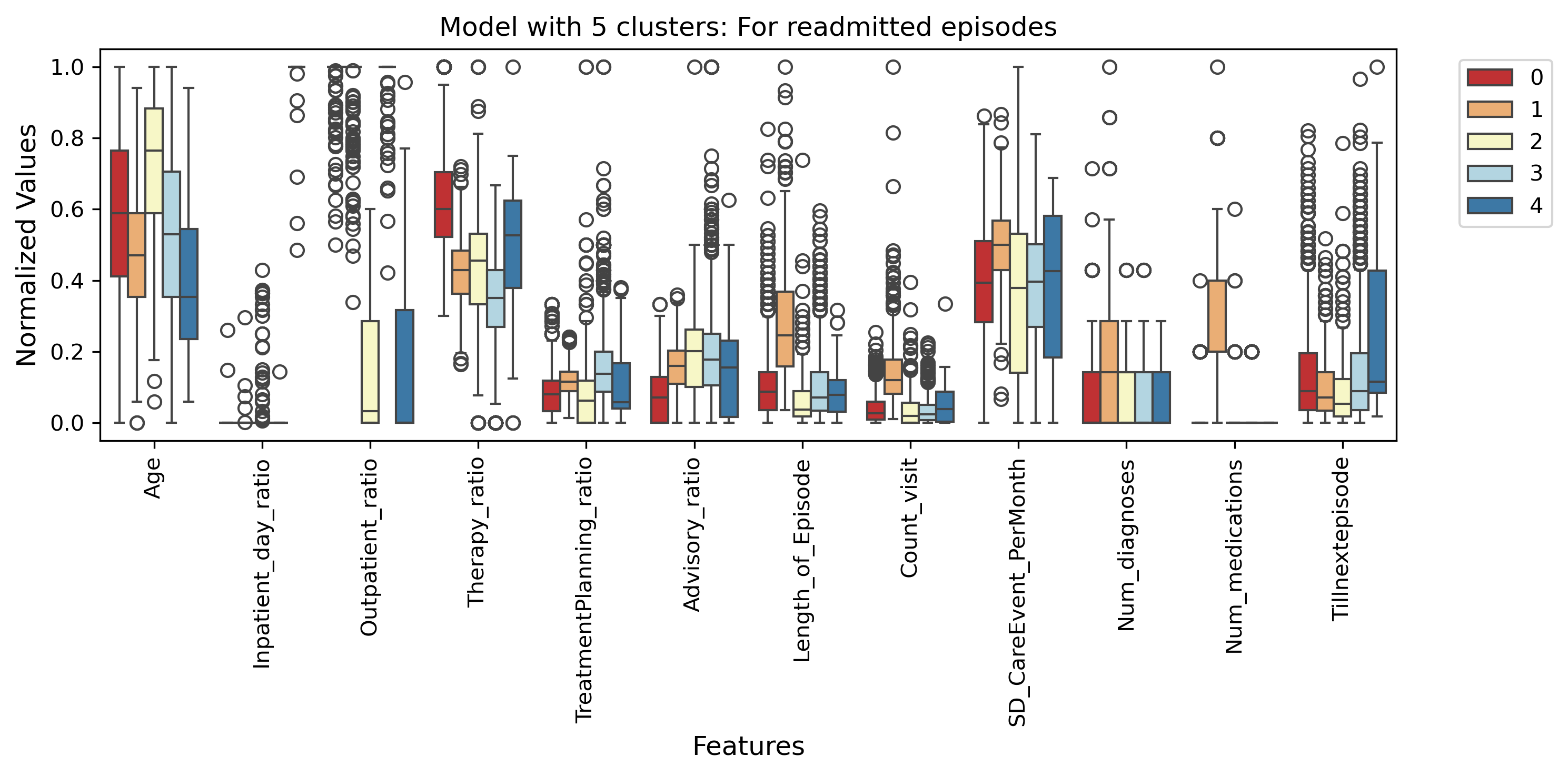

Supplement: Supplemental Information 12 [file peerj-cs-10-2367-s012.png]

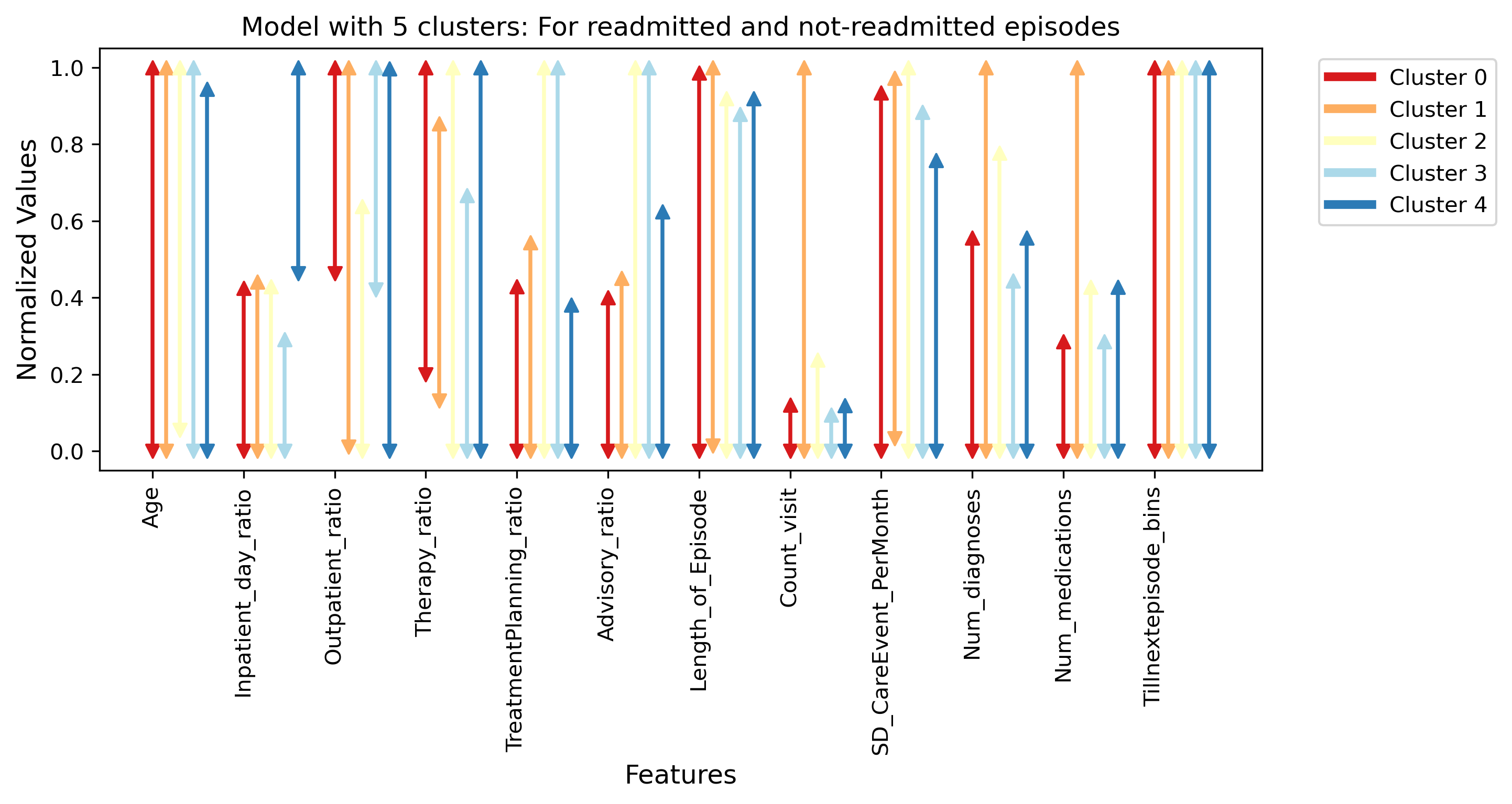

Supplement: Supplemental Information 13 [file peerj-cs-10-2367-s013.png]

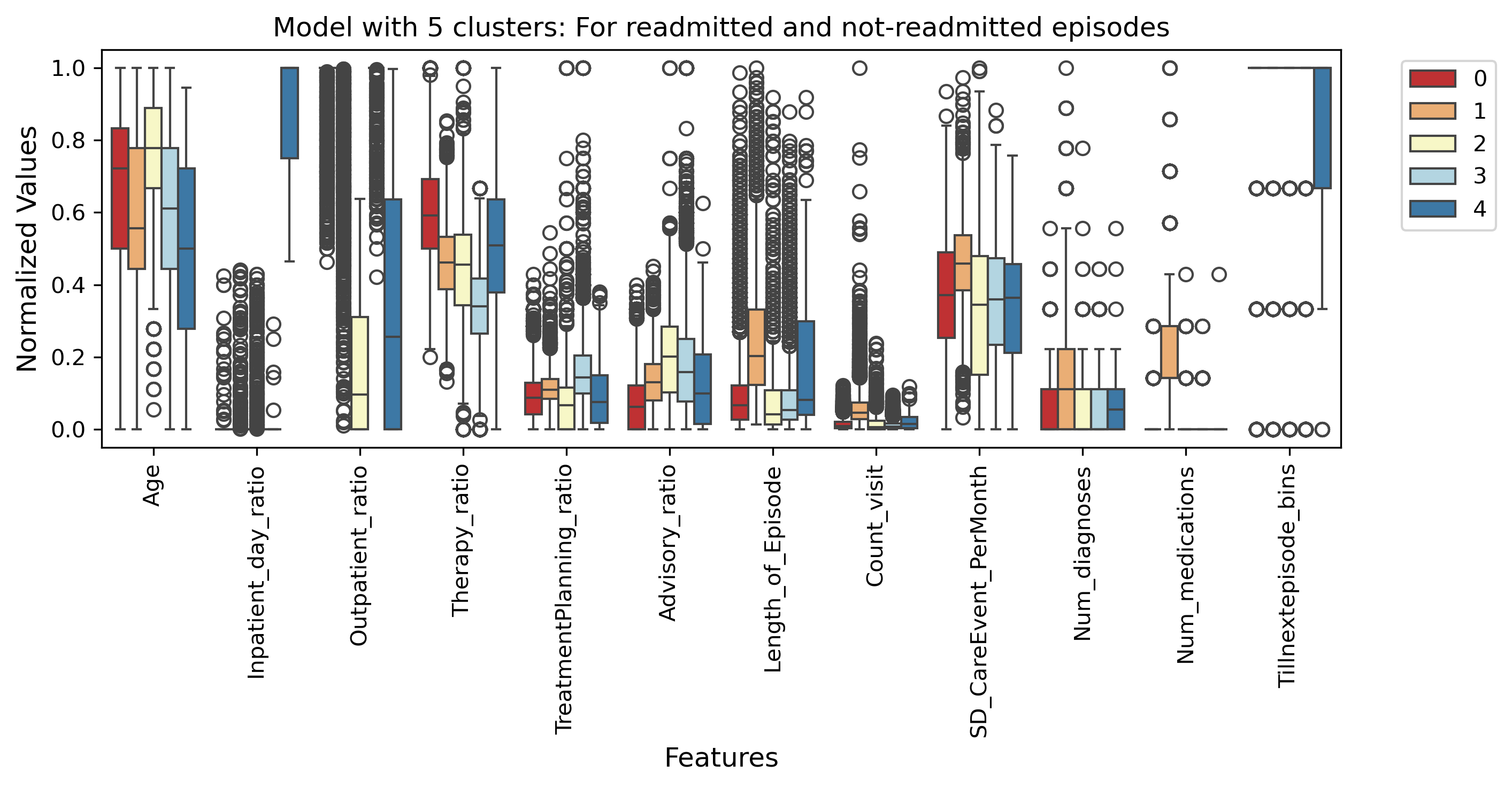

Supplement: Supplemental Information 14 [file peerj-cs-10-2367-s014.png]

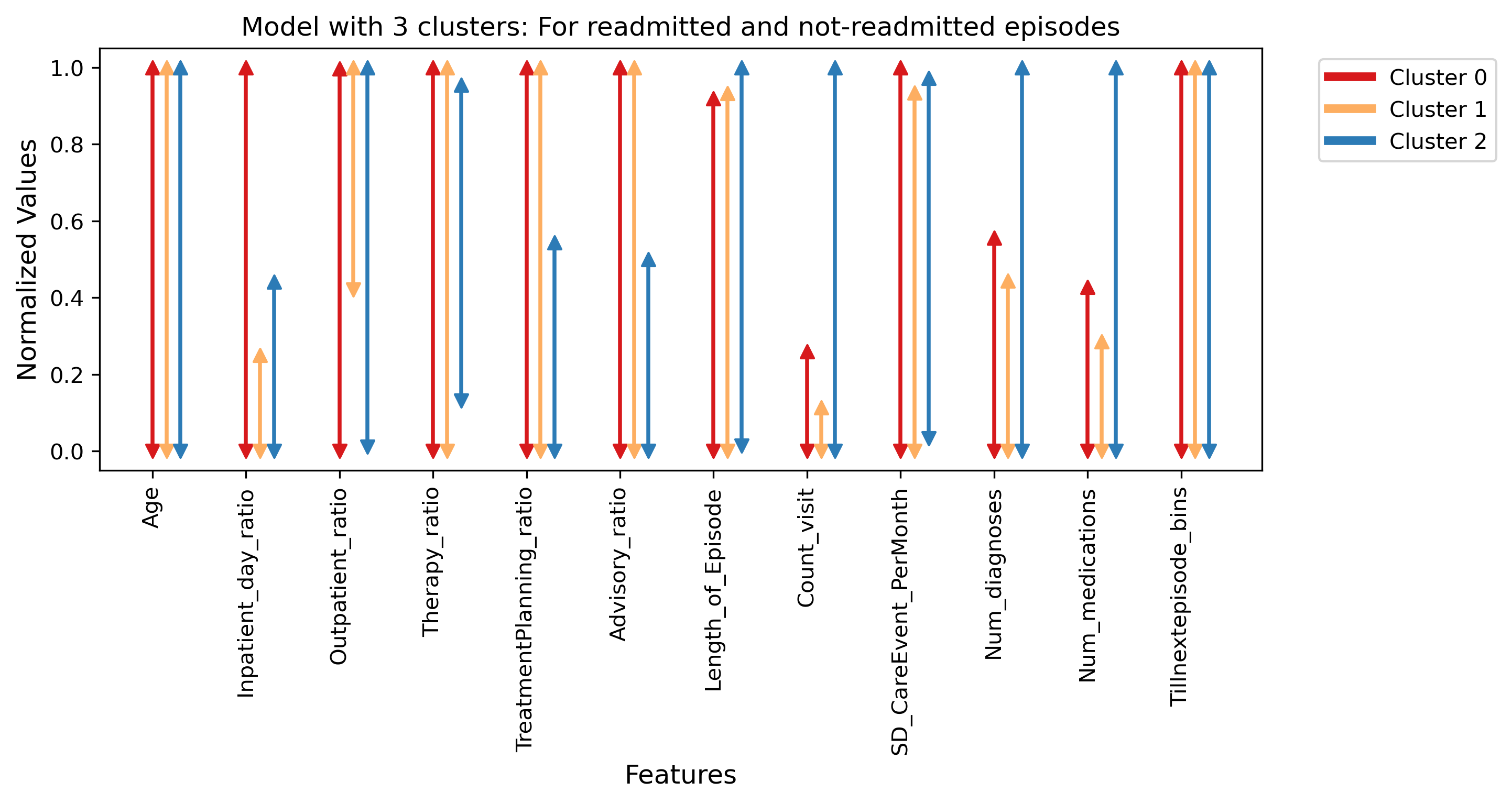

Supplement: Supplemental Information 15 [file peerj-cs-10-2367-s015.png]

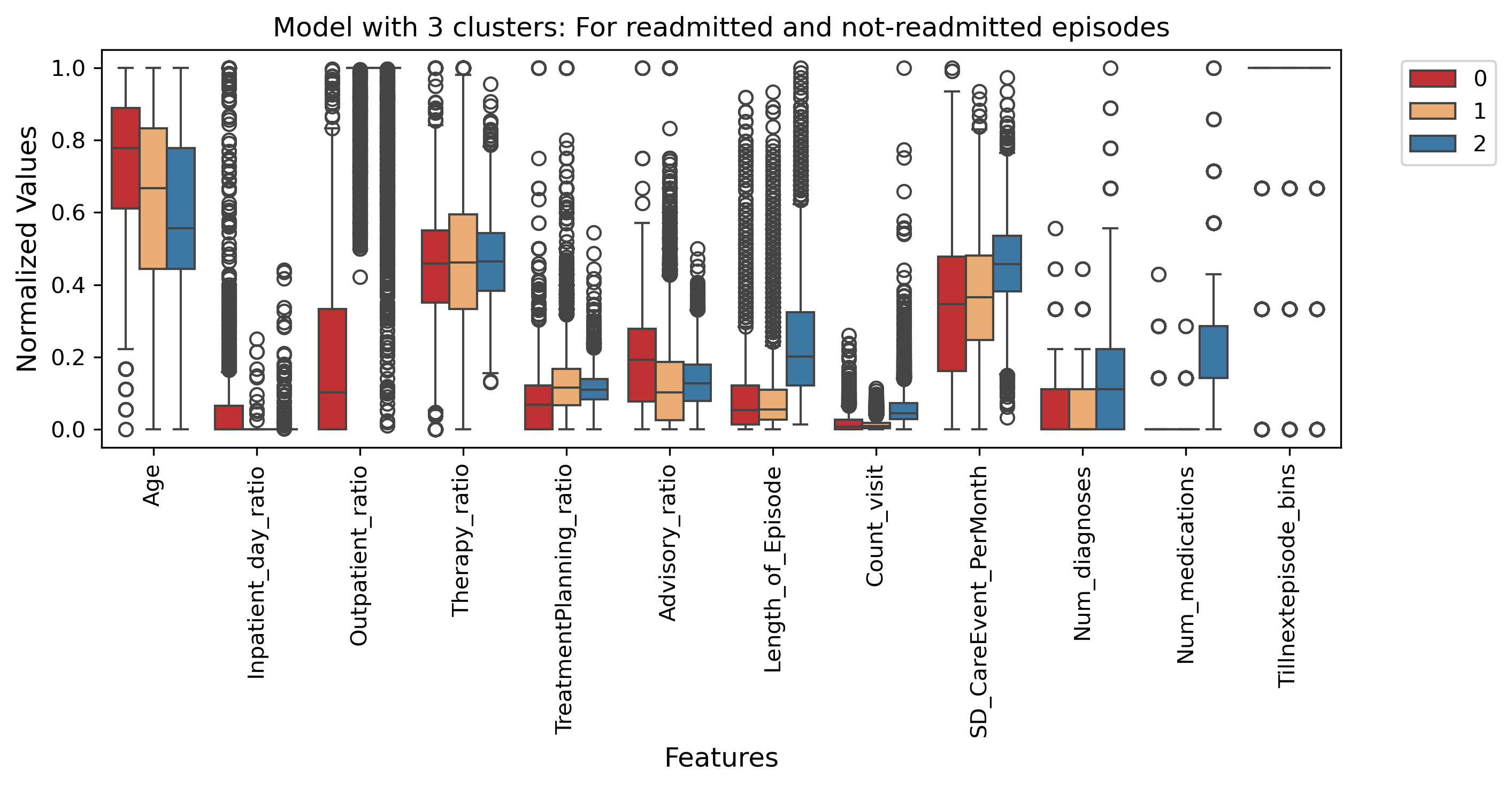

Supplement: Supplemental Information 16 [file peerj-cs-10-2367-s016.png]

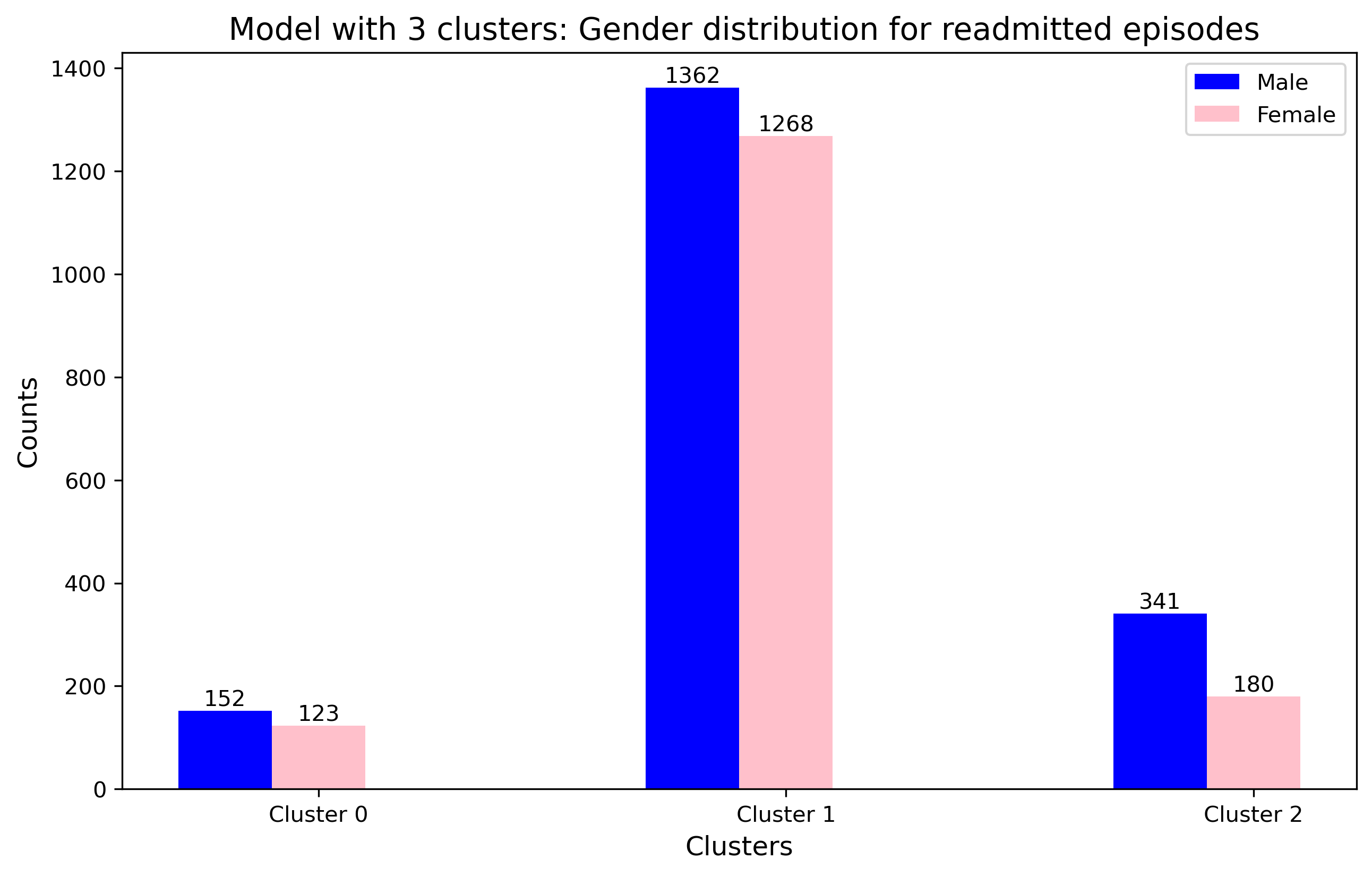

Supplement: Supplemental Information 17 [file peerj-cs-10-2367-s017.png]

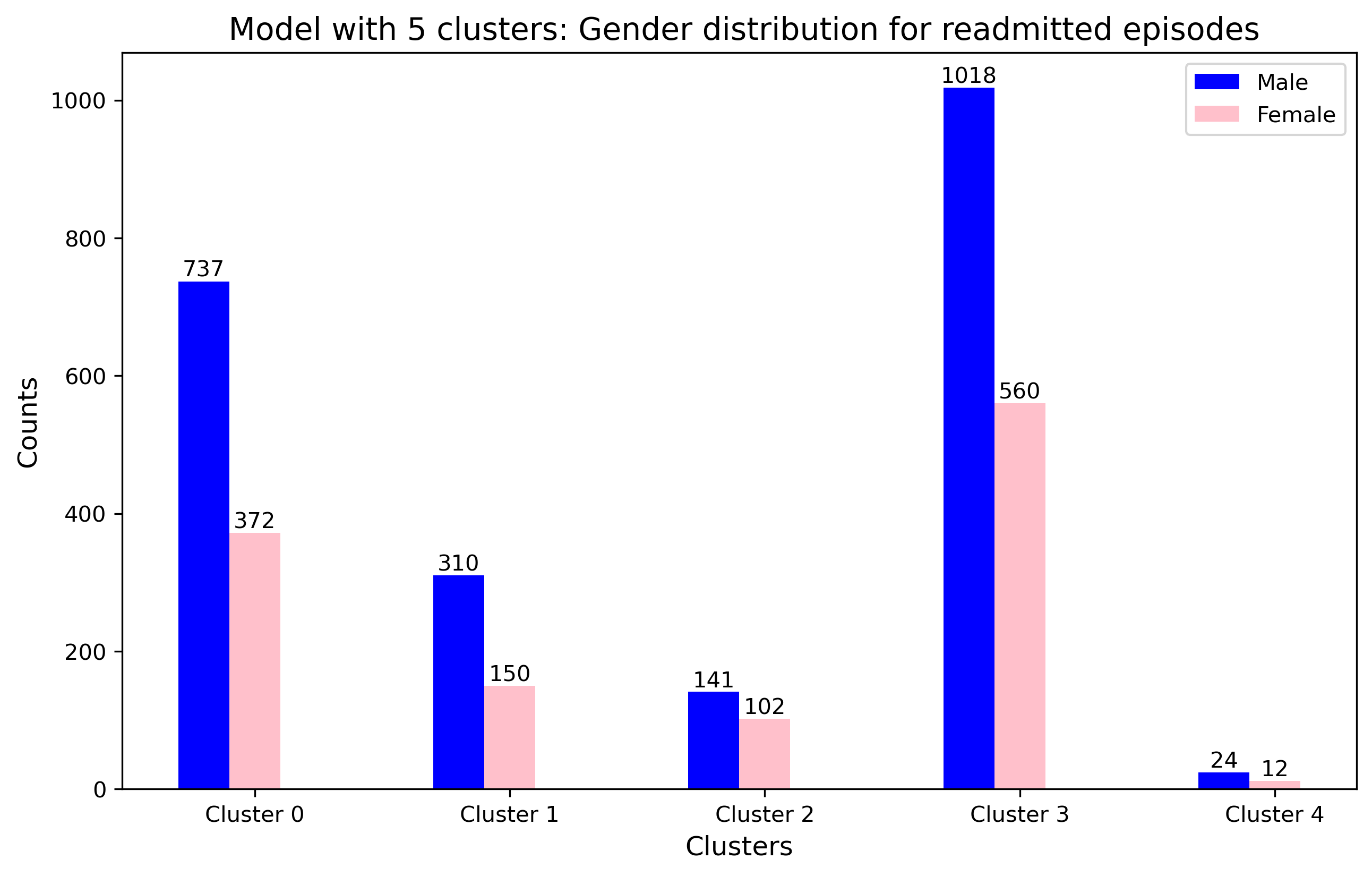

Supplement: Supplemental Information 18 [file peerj-cs-10-2367-s018.png]
