# Supplementary material for: Ability of clinical data to predict readmission in Child and Adolescent Mental Health Services
Source: PeerJ Comput Sci. 2024 Oct 18;10:e2367. doi: 10.7717/peerj-cs.2367 (PMC11622991; doi:10.7717/peerj-cs.2367)
Supplement: Supplemental Information 24 [file peerj-cs-10-2367-s024.docx]

========================================================================

### **Hyperparameters Used for Readmission Prediction**

========================================================================

#### **Binary Classifiers**

1. **DecisionTreeClassifier**:
   - random_state: 42
   - No specific hyperparameters were mentioned in the code, custom class weights were applied.
2. **LogisticRegression**:
   - random_state: 42
   - max_iter: 1000
   - class_weight: 'balanced'
3. **RandomForestClassifier**:
   - random_state: 42
   - class_weight: 'balanced'
   - n_jobs: -1 (in custom weighting)
4. **GradientBoostingClassifier**:
   - n_estimators: 100
   - learning_rate: 0.1 (in custom weighting)
   - random_state: 42
   - sample_weight: computed using custom weighting
5. **XGBClassifier**:
   - random_state: 42
   - ratio of negative to positive class (in custom weighting): scale_pos_weight=float(counts[0] / counts[1],random_state=42
6. **MLPClassifier**:
   - random_state: 42
   - max_iter: 1000
   - learning_rate: 'adaptive' (in version 2 and custom weighting)

===================================================

#### **B. Multi-Label Classifiers**

1. **DecisionTreeClassifier**:
   - random_state: 42
   - class_weight: 'balanced'
2. **LogisticRegression**:
   - random_state: 42
   - max_iter: 1000
   - class_weight: 'balanced'
3. **RandomForestClassifier**:
   - random_state: 42
   - class_weight: 'balanced'
   - n_jobs: -1 (in custom weighting)
4. **GradientBoostingClassifier**:
   - n_estimators: 100
   - learning_rate: 0.1
   - random_state: 42
   - sample_weight: computed using compute_sample_weight(class_weight='balanced', y=y_train) in version 2
5. **XGBClassifier**:
   - random_state: 42
   - sample_weight: computed using compute_sample_weight(class_weight='balanced', y=y_train) in version 2
   - scale_pos_weight: ratio of negative to positive class (in custom weighting)
6. **MLPClassifier**:
   - random_state: 42
   - max_iter: 1000
   - learning_rate: 'adaptive'
